# Predictors of family-focused practices among mental health workers in Quebec

**DOI:** 10.3389/fpsyt.2024.1380001

**Published:** 2024-05-13

**Authors:** Geneviève Piché, Aude Villatte, Marie-Ève Clément, Marie-Hélène Morin, Darryl Maybery, Andrea Reupert, Stéphane Richard-Devantoy, Marianne Fournier-Marceau

**Affiliations:** ^1^ Département de psychoéducation et de psychologie, Université du Québec en Outaouais, Saint-Jérôme, QC, Canada; ^2^ Centre de Recherche Universitaire Sur les Jeunes et les Familles (CRUJEF), Québec, QC, Canada; ^3^ Réseau de recherche en santé des populations du Québec (RRSPQ), Montréal, QC, Canada; ^4^ Unité de Formation et de Recherches (UFR) de Psychologie, Université Toulouse Jean Jaurès, Laboratoire Psychologie de la Socialisation - Développement et Travail (PSDT), Toulouse, France; ^5^ Département de travail social, Université du Québec à Rimouski, Rimouski, QC, Canada; ^6^ Department of Rural and Indigenous Health, Monash University, Melbourne, VIC, Australia; ^7^ School of Educational Psychology and Counseling, Monash University, Melbourne, VIC, Australia; ^8^ Department of Psychiatry and Douglas Mental Health University Institute, McGill Group for Suicide Studies, McGill University, Montréal, QC, Canada

**Keywords:** family-focused practice, parenting, parental mental illness, mental health professionals, children of parents with a mental illness, mental health services

## Abstract

**Context:**

Engaging family members in the ongoing care of individuals with mental illness is a practice known to bolster the client’s recovery journey and enhance the overall wellbeing of both children and families involved. Despite its potential benefits, there remains a dearth of understanding surrounding the implementation of family-focused practices (FFP) by mental health professionals serving adults, as well as the factors that could either promote or hinder such practices. This knowledge gap is particularly pronounced within North American settings.

**Goal:**

The goal of this study was to identify potential hindering and enabling factors of FFP used in adult mental health services.

**Methods:**

A sample of 512 professionals working with adult mental health clients, from all regions of Quebec, Canada, with a variety of disciplinary backgrounds and working in different work settings, completed the Family Focused Mental Health Practice Questionnaire (FFMHPQ). Multinominal logistic regression analysis was performed to assess the impact of several factors – organizational, professional, and personal – on the degree of family-based practices of mental health workers.

**Results and discussion:**

Findings of this study show that the strongest predictors for the adoption of higher FFP levels among adult mental health professionals in Quebec, are being employed on a full-time basis, perceiving a higher level of skills, knowledge, and confidence toward FFP, and having a supportive workplace environment. Results underscore the need to address both organizational and worker-related aspects to effectively promote better FFP in mental health services.

## Background

Fostering the mental health of children of parents with a mental illness, in addition to providing support to the parent with a mental illness, is recommended practice in Canada and elsewhere (1–4). Children with a parent with a mental illness (4, 5) represent a highly vulnerable group (6, 7), and are at greater risk of developing psychosocial and school adjustment difficulties, as well as mental illnesses, than other children (8–10). Additionally, some children will provide significant support to their parent, by helping them with their treatment, assuming domestic responsibilities, and providing them emotional support, often at the expense of their own needs (10, 11). Compared to their same-aged peers, children who have a parent with a mental illness report more conflictual parent-child relationships, as well as situations of verbal or physical abuse (12, 13), trauma and neglect (14, 15). Moreover, parents with a mental illness may worry about fulfilling their parenting role, when the symptoms of their mental illness interfere with their ability to meet their children’s needs (16). For example, parents report feelings of “guilt” and have perceptions of “failure as a person and as a parent” and describe a loss of hope in their parenting role (16). Parental stress has also been shown to predict high levels of depressive symptoms in mothers (17).

As nearly half of adult users of mental health services are parents (18), adult mental health professionals have an important role to play in identifying and supporting children, parents and families, and, when needed, referring them to other services (19, 20). Family focused practices (FFP) involve mental health professionals working with the client and his or her family, including children (4). In mental health services, practices may involve identifying client's children, offering information to families supporting the parent in his parenting role, proposing a support group for children, depending on the families’ needs (4, 19, 21–23). FFP has been found to reduce the risk of intergenerational transmission of mental illnesses and improve the psychosocial adjustment and mental health of children of parents with a mental illness, while promoting the mental health recovery of parents who have a mental illness (4, 24–26). Notably, the risk of developing a mental illness decreases by 40-50% when children of parents with a mental illness receive appropriate support (2, 9). Such practices form part of a promising selective prevention strategy to improve the mental health of children, parents and families at a population level (27).

Thus, there is a need for a family-focused approach in psychiatry and mental health services, that goes beyond solely working with the adult client and entails addressing the needs of the whole family. However, to date, FFP appears to be scarcely provided in adult mental health services (28–30). To illustrate, in Norway, only 56% of clients’ minor children are identified by professionals (31), and of these, only one-third have benefited from some kind of support (32).

A number of organizational, professional, and personal factors have been highlighted as enabling or inhibiting the use of FFP (33–36). First, organizational-related factors, such as perceived workplace support (37–40), has been identified as an important predictor of FFP. Coworkers can offer guidance and emotional support to mental health professionals confronted with difficult or sensible situations, while managers can give access to specific training or supervision. Some studies have explored other organizational factors such as time and workload, or the proportion of caseloads with a parental status. However, the results are still inconclusive, particularly regarding which element plays the most significant role (39, 41–44). Additionally, a high workload perception may be linked to the employment status of the professional (45), as well as the nature or complexity of the cases in their caseload (46).

Previous research has emphasized the significance of worker-related factors in predicting practices that aim to identify dependent children of adults receiving mental health services, or of practices that offer support to families. One key factor is the nature of the profession or job role, which can influence the extent to which workers engage in these practices (28, 47–49). Studies suggest that psychologists tend to engage in such practices to a lesser extent than social workers, who are more likely to identify and support families on a routine and systematic basis. In addition, some studies have looked at the impact of professionals’ attitudes towards FFP and their beliefs about the benefits of such practices for their clients and families (38, 50, 51). Results are mixed, with some studies indicating that attitudes and beliefs strongly influence FFP (51) while others have found no significant associations (50).

Furthermore, prior research underscores the pivotal role of professionals’ perceived skill and knowledge around parental mental illness and family relationships as an important enabler of FFP (38, 39, 42, 43, 52). As such, some professionals express a lack of confidence in their preparedness to assist parents with mental health challenges in navigating their parental challenges or in evaluating family needs, thereby impeding the adoption of FFP (51, 53). Nonetheless, while a perceived need for training was not significantly associated with lower scores of FFP in one study (43), receiving training in family intervention or FFP specifically was identified as a predictor of FFP uptake in various studies (41, 43, 47). Similarly, the number of years of experience has been recognized as a predictor of FFP in some instances (44, 53), although its significance was not consistently supported across all studies (e.g., 43). Consequently, the literature remains inconclusive regarding the impact of training, years of experience, and perceived proficiency on the facilitation of FFP.

Finally, certain personal characteristics of professionals may impact their use of FFP. These include being a female (41, 43), being a parent or having confidence around children (42, 44). Additionally, personal experience of mental illness may also play a role (44). However, the evidence is still scarce as very few studies have investigated these factors.

In summary, several studies have delved into the determinants influencing the adoption of FFP among mental health professionals. However, the current body of evidence presents a heterogeneous picture concerning the influence of workplace (such as support, caseload loading, and employment status), worker (including training, years of experience, and perceived competence) and personal experiential factors (such as familiarity with children or mental illness). Additionally, this study is the first to investigate predictors of FFP within North American contexts.

Thus, the aim of the current study is to investigate the practices, attitudes, knowledge, and skills related to FFP of a sample of the Quebec adult mental health professional workforce. The study also aims to identify the factors that predict the use of FFP with parents who have a mental illness and to examine their relative importance. This is important information that can be used to benchmark practices, inform training and policy and the deployment of resources. Based on previous research, it is hypothesized that professionals’ attitudes around the relevance of FFP, workplace support, and confidence about using FFP will be associated with higher FPP scores, compared to those with lower FFP scores.

## Methods

### Design

This study was approved by the Research Ethics Boards of the Université du Québec en Outaouais (#2021-1167), the CIUSSS de la Capitale-Nationale (#MP-13-2021-2135). A cross-sectional design was used to recruit a convenience sample of French-speaking Canadian adult mental health workers across Quebec (Canada). To be eligible, professionals had to meet the following criteria: 1) work at the time of the survey with adult with mental illness (under 65 years old), 2) have direct contact with clients, and 3) be fluent in French. Those working exclusively with children were excluded.

From March to December 2021, all eligible professionals were invited to read a detailed description of the study, sign a consent form and respond to an online questionnaire via a LimeSurvey platform. Following Fan and Yan (54) recommendations, various recruitment strategies were employed to ensure a satisfactory participation rate: 1) emails to professional groups and orders, 2) emails sent through managers of all integrated health and social services centers (CISSS) from each of the 17 administrative regions of Quebec, medical clinics and community organizations offering adult mental health services and 3) promotion on social networks and local newspapers. Presentations conducted by the principal investigator were delivered at targeted workplaces (13 CISSS) to explain the project and to seek assistance with the recruitment of professionals. To encourage participation, compensation prizes were drawn among study participants, with one $25 prize per 50 participants. A total of 512 French-speaking adult mental health workers completed the questionnaire.

### Measures

The study collected sociodemographic and occupational information such as gender, age, ethnicity, years of experience in mental health, workplace and location of services, employment status (full-time or part-time), percentage of parents with mental illness on caseload (low: ≥ 20%, moderate: 21-50%, high: >50%), level of education, personal experience of mental illness (yes or no), level of comfort around children.

The French version of the *Family-Focused Mental Health Practice Questionnaire* (FFMHPQ-FR) was used to collect self-report data on mental health professional’s FFP and related factors. The original questionnaire consisted of 45 items rated on a seven-point Likert scale (1=*strongly disagree* to 7=*strongly agree*) and demonstrated good psychometric properties for 13 of the 16 subscales (Cronbach’s alphas = 0.61 to 0.89) (55). The development of the FFMHPQ-FR underwent a rigorous process, as detailed in Piché et al. (56). It was initially translated into French through a back-translation procedure (57) and was subsequently adapted to improve its psychometric proprieties (i.e. seven items added to the subscales with limited consistency) and to consider recent literature (i.e. three item added on attitudes and beliefs toward FFP) (36). Finally, it was validated through both exploratory and confirmatory factor analysis, resulting in 42 items grouped into five subscales (*Family-Focused Practices; Workplace support for FFP*; *Skills, knowledge and confidence*; *Openness to improve practice*; *Attitudes and beliefs toward FFP*), with good psychometric properties (α = 0.61 to 0.89) (**Table 1**) (56). Scores are calculated by averaging the items included within each subscale for every participant.

**Table 1:** FFMHPQ-FR and average subscale’s scores among participants.

Items by subscale		α	Average score (SD)
**Family-focused practices** *Professionals’ perceived level of family-focused practices (FFP) provided to clients and their families.*		0.85	5.10 (0.95)
1	In my practice, I instinctively check if the mentally ill adult I’m working with has a child or children (aged 0 to 25 years).		6.28 (1.14)
2	I regularly check whether parent-users are able to meet the socio-emotional needs of their children.		5.80 (1.20)
3	I provide written materials (e.g. education, information) about parenting to parent-users.		5.49 (1.40)
4	I regularly provide information (including written materials) about mental health issues to the children of parent-users with whom I work.		4.20 (1.68)
5	I do meet with parents and families in the course of my employment (not family therapy).		4.28 (1.65)
6	I regularly discuss strategies with parent-users to strengthen their parenting practices or better meet the needs of their children.		5.09 (1.47)
7	I regularly refer the children of parent-users to specific support services.		5.22 (1.42)
8	I regularly refer parent-users to parent-related programs (e.g. parenting skills).		4.85 (1.59)
9	I regularly refer the partners of parent-users to specific support services.		4.61 (2.00)
**Workplace support for FFP** *Professional’s perceived support from their workplace (e.g. resources, supervision) to engage in FFP.*		0.87	3.69 (1.03)
10	My workplace provides supervision to support professionals who help users with their parenting role.		3.30 (1.81)
11R	In my workplace, there is not enough time to work with families or children of parent-users.		3.21 (1.70)
12R	Professional development regarding family centered practice is not encouraged at my workplace.		4.43 (1.69)
13	I often receive support from co-workers in regard to family centered practice.		
18R	It is difficult to refer parent-users to parenting support programs (e.g., absence, non-accessible).		3.26 (1.53)
20R	My workplace does not provide supervision to support professionals undertaking family centered practices.		3.44 (1.80)
21R	My workload is too high to do family centered work.		3.37 (1.68)
22R	My workplace provides little support for professional development in family centered practices.		3.29 (1.68)
23	In my workplace, other professionals encourage family centered practice.		4.92 (1.58)
28R	It is difficult to refer families of parent-users to family therapy or family counselling services (e.g., absence, non-accessible).		3.04 (1.57)
30	At my workplace, we have time to establish regular contact with partner organizations concerning families or parent-users.		3.76 (1.79)
40R	In my workplace, my professional mandate does not allow me to use a family-centered practice		4.10 (1.91)
**Skills, knowledge and confidence in FPP** *Professionals’ perceived skills, knowledge, and confidence in undertaking FFP.*		0.89	4.82 (0.97)
14R	I am not confident working with parent-users about their parenting skills.		4.70 (1.93)
15	I am able to determine the developmental progress of the children of parent-users with whom I work.		4.03 (1.64)
17	I am knowledgeable about how parental mental illness impacts on families.		5.42 (1.23)
19	I am able to assess the importance that parent place on their children participation in social and recreational activities.		5.12 (1.36)
24R	I do not have enough confidence in my professional skills to work with the families of parent-users.		4.96 (1.63)
25	I am able to assess the extent to which the manifestations of mental illness of parent-users have an impact on their children.		5.16 (1.28)
27R	I do not have the skills to help parent-users recognize the possible impact of their mental illness on their children and families.		4.70 (1.64)
29	I am able to determine the importance that parent-users place on maintaining good relationships between their children and other family members.		5.53 (1.15)
35R	I have no experience in working with the children of parent-users.		4.16 (1.90)
36R	I am not able to assess the importance that parent-users place on the quality of the support network (outside the family) of their children.		4.92 (1.53)
38	I am skilled to support parent-users in promoting the well-being of their children.		4.91 (1.36)
41R	I do not have enough confidence in my professional skills to work with the children of parent-users.		4.66 (1.75)
42	I know the main techniques that parent-users could use to promote the well-being of their children.		4.39 (1.56)
**Openness to improve FFP** *Professionals’ intention to engage in training and improve their knowledge and skills in FFP.*		0.82	5.60 (0.95)
16	I sometimes wish that I was better able to help parents discuss the impact of their mental illness on their children.		5.72 (1.19)
26	I should learn more about how to assist parent-users about their parenting role.		5.48 (1.34)
33	I would like to undertake future training to improve my professional skills to work with the children of parent-users.		5.78 (1.35)
37	I would like to undertake training to improve my professional skills to support users in their parenting role.		5.89 (1.25)
39	I would like to better understand my role to support families, as a professional within a care team.		5.11 (1.47)
**Attitudes and beliefs toward FFP** *Professionals’ attitudes and beliefs regarding the relevance of incorporating FFP in their practice.*		0.61	6.00 (0.84)
31	No matter the level of severity or type of illness of the parent-users, use of a family centered practice is always relevant.		5.63 (1.31)
32R	Use of a family-centered practice is irrelevant when there is no risk of violent or suicidal behavior in a parent-user.		6.36 (0.93)
34	Adopting a family-centered practice is always relevant, regardless of the age of the children of the parent-users.		6.01 (1.12)

### Data analysis

Across the 42 items of the FFMHPQ-FR, the rate of valid data amounted to 90.8%. Participants were required to answer each question in the questionnaire, while also having the option to select the responses “refusal to answer” or “does not apply.” These response choices were coded as missing data and treated using mean imputation per item (58).

All statistical analyses were performed using SPSS Version 27.0 software. As a preliminary analysis, the following assumptions were tested: sample size, multicollinearity, outliers and normality. Outliers were treated by winsorizing 2^nd^ and 98^th^ percentile. Descriptives (means, standard deviations) were calculated for all variables. Analyses of variance (ANOVA) and chi-square tests were conducted aiming to verify the relationship between mental health worker’s FFP and variables of interest in this study. *Post-hoc* analyses allowed to specify the nature of the differences between the groups.

A multinomial logistic regression analysis was performed to assess prediction of mental health worker’s FFP on the basis of ten factors: *Workplace support for FFP* subscale, employment status, percentage of parents with mental illness on caseload, years of experience in mental health, level of education, *Skills, knowledge and confidence* subscale, *Openness to improve practice* subscale, *Attitudes and beliefs toward FFP* subscale, level of comfort around children, and personal experience of mental illness. To perform this, scores from the *FFP* subscale were recoded into three categories: low (1.00-4.77), moderate (4.78-5.55) and high (5.56-7.00) score of FFP, based on percentile-based cut-points (respectively on the 33^rd^ and 67^th^ percentiles). Using a three-categorical variable will provide interpretable coefficients to quantify the relationship between predictors and the outcome variable. Thus, the high score category allows to discriminate professionals that provide more FFP to clients and their families. The results are presented as inverted odds ratio (IOR), easier to interpret with negative beta. The significance level in all analyses was .05.

## Results

### Participants

The majority of participants were female (87.1%) and the average age was 40.34 years (range 19-75 years). Most were Caucasians (95.9%), born in Quebec (92.0%). The majority (85.7%) were university graduates, from various disciplines including social work (27.9%), nursing (20.9%), psychology (13.9%), psychoeducation (13.1%) and special education (8.0%). The average number of years’ of experience in mental health was 11.77 (range 1-40 years). Among the participants, 27% reported that more than half of their caseload included clients with mental illness who have at least one minor child. In the past five years, 43.3% had received family-focused training, while 34.8% had attended child-focused training.

**Table 1** presents the average scores of participants on the FFMHPQ-FR’s subscales and items. The average score on the *FFP* subscale (M=5.13; SD=1.06) indicates a moderate level of FFP among mental health professionals in this sample. The highest item scores were reported on *Identification of children*, *Evaluating parenting competencies* and *Information to parents*, while the lowest were attributed to *Information to children*, *Family meetings* and *Referrals to support services*. Among the subscales, the participants scored the lowest on *Workplace support for FFP* (M=3.70; SD=1.13), with most items scoring under 4. The highest subscale scores were reported on the *Attitudes and beliefs toward FFP* (M=5.58; SD=1.04) and the *Openness to improve FFP* (M=6.00; SD=0.85).

**Table 2** presents descriptive statistics of all variables, according to the three percentile-based categories of FFP score (low, 34.8%; moderate, 32.4%; high, 32.8%). Differences between groups for the following eight independent variables have been identified through post-hoc analyses: *Workplace support for FFP*, employment status, proportion of parental clientele, *Skills, knowledge and confidence in FFP*, *Openness to improve FFP*, *Attitudes and beliefs toward FFP*, highest academic degree, and years of experience in mental health.

**Table 2:** Comparisons of variables of interest on Family-Focused practice score.

Variables			Family-Focused Practices						P-value	Post-hoc
	Total (n=512)		Low score (n=178)		Moderate score (n=166)		High score (n=168)			
Continuous	M	ET	M	ET	M	ET	M	ET		
Workplace support for FFP	3.69	1.03	3.32	0.87	3.71	0.97	4.08	1.11	<.001	LS < MS < HS
Skills. knowledge and confidence in FFP	4.82	0.97	4.45	0.99	4.77	0.88	5.26	0.86	<.001	LS < MS < HS
Openness to improve FFP	5.60	0.95	5.60	0.97	5.61	0.88	5.61	0.99	0.986	
Attitudes and beliefs toward FFP	6.00	0.84	5.82	0.87	5.92	0.83	6.27	0.75	<.001	LS, MS < HS
Percentage of caseload with a parental status	1.91	0.79	1.80	0.76	1.86	0.79	2.07	0.82	0.006	LS < HS
Highest academic degree	3.36	0.99	3.56	1.15	3.25	0.93	3.24	0.82	0.002	HS < LS, MS
Years of experience in mental health	11.77	9.10	10.08	8.75	11.61	8.53	13.72	9.66	0.001	LS < HS
Level of comfort around children	5.93	1.15	5.66	1.21	5.97	1.15	6.19	1.01	<.001	LS < MS. HS
Categorical	N	%	N	%	N	%	N	%		
Employment status									0.004	
Full-time	430	84.6	142	80.2	134	81.7	154	92.2		LS, MS < HS
Part-time	78	15.4	35	19.8	30	18.3	13	7.8		HS < LS, MS
Total	508		177		164		167			
Personal experience of mental health									0.269	
Yes	341	68.1	86	62.8	140	71.1	115	68.9		
No	160	31.9	51	37.2	57	28.9	52	31.1		
Total	501		137		197		167			

The number of participants may vary due to missing data. Only valid data is reported considering that the data collected includes a negligible number of missing data for the sociodemographic characteristics (1.0%). Some terms have been abbreviated: LS, low score; MS, moderate score; HS, high score.

### Predictors of FFP

The model including all ten predictors, when compared to a constant-only model, was statistically significant, χ2 (5, N=482) = 145.22, p<.001. This indicates that the predictors, as a set, significantly discriminate between low, moderate, and high score participants. The model explained 29.3% of the variance in FFP score, according to Nagelkerke R².

This model classified 53.3% of cases correctly, which is greater than the proportional by chance criterion of 41.7% (**Table 3**). However, specific classification rates (low score, 66.1%; moderate score, 28.4%; high score, 64.2%) underline the model’s tendency to under classify the moderate FFP scores compared to low and high score categories.

**Table 3:** Multinomial logistic regression results – Classification table.

Family-focused practices	Real N (%)	Proportional by chance criterion	Predicted N (%)
Low score	165 (34.2%)	11.7%	109 (66.1%)
Moderate score	155 (32.2%)	10.3%	44 (28.4%)
High score	162 (33.6%)	11.3%	104 (64.2%)
Global percentage	100.0%	41.7%	53.3%

Comparing low score FFP with high score FFP categories, it was found that the strongest predictors of reporting a high FFP score as a professional are employment status (full versus part time) (IOR=2.34), and skills, knowledge, and confidence toward FFP (high versus low score) (IOR=2.26) (**Table 4**). This indicates that professionals employed full-time are 2.34 times more likely to report a high FFP score rather than a low FPP one, controlling for all other factors in the model. Likewise, for every point increase on the *Skills, knowledge and confidence* subscale, professionals are 2.26 times more likely to present a high FFP score. The perceived level of workplace support for FFP (high versus low) (IOR = 1.90), openness to improve FFP (high versus low) (IOR = 1.52), and attitudes and beliefs towards FFP (high versus low) (IOR =1.61) were also found as important predictors of FFP. Weaker predictions were found between high FFP and the proportion of caseloads with a parental status (high versus low) (IOR = 1.49) and the professional’s years of experience working in mental health (high versus low) (IOR = 1.04). The professional’s education (highest diploma), being comfortable with children, and personal experience with mental illness were not found to predict a high score of FFP compared with a low FFP score. 

**Table 4:** Multinomial logistic regression results – High FFP score compared to low and moderate FFP scores.

Variables	Categories of outcome					
	Low score (n=165)			Moderate score (n=155)		
	ß	OR (95% CI)	IOR	ß	OR (95% CI)	IOR
Organizational factors						
Workplace support for FFP	-0.64***	0.53 (0.40-0.69)	1.90	-0.23	0.79 (0.62-1.01)	1.26
Employment status	-0.85*	0.43 (0.19-0.94)	2.34	-0.81*	0.44 (0.21-0.94)	2.26
Proportion of clientele with a parental status	-0.40*	0.67 (0.49-0.93)	1.49	-0.28	0.76 (0.56-1.02)	1.32
Professional factors						
Experience working in mental health	-0.04*	0.96 (0.94-0.99)	1.04	-0.02	0.98 (0.96-1.01)	1.02
Highest academic degree	-0.23	1.26 (0.95-1.66)	0.80	-0.02	1.02 (0.77-1.34)	0.98
Skills, knowledge and confidence in FFP	-0.82***	0.44 (0.32-0.60)	2.26	-0.50**	0.61 (0.45-0.82)	1.65
Openness to improve FFP	-0.42*	0.66 (0.48-0.91)	1.52	-0.20	0.82 (0.61-1.10)	1.23
Attitudes and beliefs toward FFP	-0.48**	0.62 (0.44-0.87)	1.61	-0.46**	0.63 (0.46-0.87)	1.59
Personal factors						
Level of comfort around children	-0.23	0.79 (0.62-1.00)	1.26	-0.07	0.94 (0.74-1.19)	1.07
Personal or familial experience of mental illness	-0.27	0.76 (0.44-1.30)	1.31	-0.18	1.20 (0.71-2.03)	0.83

The reference category is = high score (n=162). * p<.05; ** p<.01; *** p<.001.

When comparing moderate FFP score with high FFP score categories, the strongest predictor was found to be the employment status of professionals (full-time versus part-time) (IOR=2.26). Thus, professionals who work full-time are 2.26 times more likely to have a high FFP score than a moderate one, controlling for all other factors in the model. The perceived skills, knowledge, and confidence in FFP (IOR=1.65) and attitudes and beliefs towards FFP (IOR=1.59) were also found to be important predictors of FFP. This indicates that for every point increase on the *Perceived skills, knowledge and confidence* and the *Attitudes and beliefs toward FFP* subscale, professionals are respectively 1.65 times and 1.59 times more likely to have a high FFP score rather than a moderate one. None of the other factors were found to be associated with a high score of FFP compared with a moderate FFP score.

## Discussion

The present study investigated the FFP of a sample of Quebec adult mental health professionals, FPP predictors and the relative importance of these predictors. Our study addressed a notable gap in the literature by investigating the use of FFP among mental health professionals in North American settings, as well as the factors influencing their engagement in such practices. By doing so, the study contributes to bolstering the evidence base in this critical area of research.

### FFP among Quebec adult mental health professionals

The study’s findings shed light on the state of FFP among mental health professionals in Quebec, revealing a generally moderate level of engagement with families. This involves actions such as checking if clients have minor children, providing written materials on mental health and parenting, and discussing parenting strategies. Moreover, professionals reported a moderate level of perceived skills, knowledge and confidence around FFP. Results also suggest that most workers understand the relevance of offering support to children and families whose parent has a mental illness, and that they are generally open to improve their practice to better support families. Interestingly, the results seem to contrast with international studies that show lower self-reported FFP levels among adult mental health workers than FFP scores in our study. For example, in Skogøy et al. (43), the average scores for the FFP subscales are respectively 3.85 (*Family support*) and 4.08 (*Referrals*). This could suggest a unique context in Quebec where professionals appear more inclined towards FFP practices.

It is however worth noting that even though the participants in this study reported using moderate FFP levels, there were variations in responses across specific FFP activities. While a majority of the sample (84%) report that they routinely inquired about clients having minor children, discussing parenting strategies and providing mental health information directly to clients’ children were not frequently reported. These findings emphasize the need for improvement in mental health care practices concerning support for children of parents with mental illness. Professionals should not only identify such children but also play a proactive role, including offering age-appropriate information on mental illness, offering help around parenting issues and referring children and parents to appropriate support services. The results underscore the importance of expanding professionals’ awareness of their role in supporting families affected by mental illness, urging them to go beyond identification and incorporate further comprehensive strategies.

Simultaneously, results indicate that workplace support scored the lowest among all the other worker or workplace factors reported by participants. This finding generally converges with the literature, underlining that few organizations have clear guidelines supporting a family-focused approach and that most professionals feel that there are still major obstacles in their workplace for them to adopt FFP with their caseload, such as lack of necessary space and resources to invite their clients’ children for family meetings, as well as limited time to do so (36, 59). Our findings thus show that Quebec professionals feel unsupported by their work environment when it comes to offering support to families of parents receiving mental health services. Most professionals report not having sufficient time to work with families, indicated that their workload is too high, and that there are no specific parenting or family programs to refer clients’ families to when needed.

### Predictors of FFP

The study revealed significant predictors of FFP, encompassing both organizational and professional factors. Surprisingly, neither of the two personal factors examined—comfort with children or personal experience with mental illness—emerged as significant predictors of professionals’ FFP scores. This finding contrasts with previous research findings (44), thus enriching the existing literature. Additionally, while some predictors were consistent across high versus low FFP scores and high versus moderate FFP scores, a closer examination reveals a greater number of significant associations between low and high FFP levels.

### Worker-related predictors

Results underscore the significance of professionals’ attitudes toward FFP, and their perceived competence, knowledge, and confidence in predicting higher levels of self-reported engagement in FFP. These findings align with prior research, emphasizing that professionals who exhibit confidence in using FFP and acknowledge its importance are more likely to integrate it into their daily practice (39, 43, 52). Recognizing the unique parenting challenges faced by parents that have a mental illness, understanding the needs of their children, and feeling confident in engaging with and offering them support, are identified as crucial elements for the successful adoption of FFP with parents and their families. Notably, participants reported moderate to high scores on these factors, indicating a positive trend toward FFP.

Two additional professional characteristics emerged as significant predictors of FFP adoption: years of experience in adult mental health and professionals’ openness to improving their practice. This aligns with previous findings, showing that professionals with more than five years of experience were more likely to report higher levels of family and parenting support than their less-experienced counterparts (41). Hence, these worker-related characteristics play a pivotal role in ensuring the adoption of FFP in their interactions with clients. On this basis, it is recommended to offer targeted FFP training to professionals, to empower them to feel confident and prepared to use FFP with parents and families (41). Specifically, such training should include informing professionals about the impact of mental illness on parenting and equipping them with practical skills for engaging, communicating, and offering support to families (60). Best practices for interacting with children and families in the context of adult mental health should be systematically integrated into treatment protocols.

### The importance of the workplace

Results show that three workplace-related factors significantly predict a high FFP score: perceived workplace support for FFP, the professional’s employment status and the percentage of their clients with children. These findings converge with earlier research (43, 52), underlining the crucial role of workplace factors, including time constraints, workload, and access to supervision, as important predictors of FFP engagement among adult mental health professionals. These findings support the importance of raising awareness among responsible for clinical programs in mental health services and community organizations, about professionals’ roles and responsibilities in FFP and the need to provide adequate resources, particularly time.

Moreover, our finding regarding employment status, revealing that full-time work basis predicts a higher level of FFP, has not been yet addressed in the literature. It is possible to hypothesize that professionals employed on a full-time basis may have more time to work with families and may benefit from enhanced training or supervision, compared to those working part-time. This is an important finding, especially considering that more and more professionals work part-time in industrialized countries (61, 62). While this could account for their higher level of FFP, future research should investigate the differences between professionals working full-time and part-time, specifically around workload, training, and supervision. Furthermore, recognizing that working with families is complex and may require more time, organizations might adjust caseload expectations when screening and assessing the needs of clients with children, as well as ensure that all professionals, regardless of their employment status, receive comprehensive support in FFP. 

To further support professionals in adopting FFP, the implementation of clear clinical guidelines and procedures is recommended. Additionally, providing access to specific FFP supervision or facilitating multi-disciplinary team discussions, might enhance understanding and acceptance of FFP as an integral part of all professions within the team (43). These measures may not only contribute to a shared sense of responsibility within the team but also help professionals feel actively involved and supported in embracing FFP practices (36). Overall, fostering a supportive work environment with the necessary resources and infrastructure is crucial to encouraging widespread adoption of FFP among mental health professionals.

Findings underscore the importance of ongoing monitoring and benchmarking of FFP within every workplace. To cultivate a work environment conducive to FFP, it is crucial for managers responsible for clinical programs to not only be aware of professionals’ roles and responsibilities but also to continually monitor the integration of FFP practices. Regular assessments and benchmarking can provide valuable insights into the effectiveness of existing support systems, allowing for necessary adjustments and improvements (63).

### Limits and recommendations for future research

Although the sample size is adequate and the profile of participating professionals is comparable to that of other studies conducted in Quebec (e.g., 64), it is nevertheless a convenience sample and may not be representative of the population of professionals working with adults receiving mental health services in Quebec. It is possible that those who participated were more concerned about children who have a parent with a mental illness, which could have led to a bias in the representativeness of the results, resulting in a tendency to overestimate their use of FFP, as evoked by Gregg et al. (36). Future studies should be conducted with a larger and more diverse sample that is representative of all professionals working with adults receiving mental health services, to enhance the generalizability of findings. It is also important to remember that the data are self-reported, questionnaire-based, and may not reflect the actual practice of professionals. More studies including direct observations or audits of professionals’ interactions with families and children (32), or qualitative interviews, should be conducted to ensure the accuracy of results, as well as longitudinal studies to track professionals’ practices over time. Finally, this study is cross-sectional, indicating that the relationships identified in our analysis may not be causative. Based on our results, it is not possible to determine whether the consideration of these predictors in the care and services provided to adults with a mental illness and their families might impact their recovery and well-being.

## Conclusion

Given the crucial role of interpersonal relationships and social networks in individual recovery, it is imperative for all mental health professionals to adopt a family-focused approach. This approach involves systematically considering the involvement of children in their interventions. Despite a wealth of evidence supporting family involvement in mental health (65), achieving systematic implementation remains challenging. Findings of this study show that the strongest predictors for the adoption of higher FFP levels among adult mental health professionals in Quebec, are being employed on a full-time basis, perceiving a higher level of skills, knowledge, and confidence toward FFP, and having a supportive workplace environment. Results underscore the need to address both organizational and worker-related aspects to effectively promote better FFP in mental health services. This study significantly contributes to the existing literature by facilitating global learning and knowledge transfer on predictors of FFP, improving understanding of global standards in mental health. Understanding the predictors for adopting family-focused practices can facilitate the development of more effective strategies and interventions that can be implemented globally.

## Data availability statement

The raw data supporting the conclusions of this article will be made available by the authors, without undue reservation.

## Ethics statement

This study was approved by the Research Ethics Boards of the Université du Québec en Outaouais (#2021-1167), the CIUSSS de la Capitale-Nationale (#MP-13-2021-2135). The studies were conducted in accordance with the local legislation and institutional requirements. The participants provided their written informed consent to participate in this study.

## Author contributions

GP: Conceptualization, Formal analysis, Funding acquisition, Investigation, Methodology, Project administration, Resources, Supervision, Validation, Visualization, Writing – original draft, Writing – review & editing. AV: Conceptualization, Funding acquisition, Investigation, Methodology, Validation, Visualization, Writing – review & editing. M-EC: Conceptualization, Investigation, Methodology, Validation, Writing – review & editing. M-HM: Conceptualization, Investigation, Methodology, Validation, Writing – review & editing. DM: Validation, Writing – review & editing. AR: Writing – review & editing. SR-D: Methodology, Writing – review & editing. MF-M: Writing – original draft, Writing – review & editing.
